# The Effects of Glucagon-Like Peptide-1 (GLP-1) Receptor Agonists on Polycystic Ovarian Syndrome: A Scoping Review

**DOI:** 10.7759/cureus.93104

**Published:** 2025-09-24

**Authors:** Mia Hudanich, Shannon N Smith, Amanda Marino, Suzanne I Riskin

**Affiliations:** 1 Department of Foundational Sciences, Nova Southeastern University Dr. Kiran C. Patel College of Osteopathic Medicine, Clearwater, USA

**Keywords:** glp-1 receptor agonist, hyperandrogenism, infertility disorders, insulin resistance, metabolic syndrome, obesity, polycystic ovarian syndrome, retatrutide, semaglutide, tirzepatide

## Abstract

Polycystic ovarian syndrome (PCOS) is the most common endocrinopathy in reproductive-aged women worldwide; however, treatment modalities often lack cohesion due to its multifactorial pathophysiology. PCOS is suspected of inducing insulin resistance. Research has explored the use of newly developed incretin mimetics as standard therapy for insulin resistance in insulin-dependent tissues associated with PCOS. The aim of this review was to explore the classes of incretin mimetics, such as glucagon-like peptide-1 (GLP-1) receptor agonists or semaglutide, dual agonists of the GLP-1 receptor and gastric inhibitory peptide (GIP) or tirzepatide, and a new triple agonist, or retatrutide (which is currently seeking Food and Drug Administration (FDA) approval), and their suggested benefits as a treatment for PCOS. A literature review was conducted using EBSCO Medline and PubMed following the Preferred Reporting Items for Systematic Reviews and Meta-Analyses (PRISMA) guidelines. All three classes of incretin mimetics showed significant improvement in weight loss and insulin sensitivity when compared to traditional pharmacological management with metformin and estradiol-progesterone combination pills in patients with PCOS. The added upregulation of GIP in dual-acting and triple-acting agonists, such as tirzepatide and retatrutide, respectively, resulted in greater reductions in weight loss and insulin sensitivity when compared to medications that acted at the GLP-1 receptor alone. Some research demonstrated symptom improvements specific to PCOS presentation, such as dysmenorrhea and the classic dysmorphic ovarian morphology. Further research is warranted to determine the exact mechanism behind how incretin mimetics may improve the hormonal dysregulation in patients with PCOS, as well as how to best use these medications in conjunction with the current standard of care treatments.

## Introduction and background

Worldwide, 5%-10% of women are diagnosed with polycystic ovarian syndrome (PCOS) annually, the most common endocrinopathy in reproductive-aged women [1]. The 2003 Rotterdam criteria, the most widely accepted PCOS diagnostic criteria in clinical practice, confirm a diagnosis of PCOS with the presence of two out of three of the following: oligomenorrhea, hyperandrogenism, or polycystic-appearing ovarian morphology on ultrasound [2]. PCOS is a multifactorial disease and is often difficult to treat due to its etiology arising in the endocrine system, with clinical manifestations in the ovaries. Because of this, treatment often requires acknowledgment of both the endocrine and gynecological systems and involves a multidisciplinary team of gynecologists, endocrinologists, and primary care providers. This often results in delayed diagnosis, as it can become difficult to develop consistent treatment plans with multiple providers involved. Inconsistent treatment guidelines only worsen the problem; appropriate treatment plans from an endocrinological viewpoint often neglect the gynecological manifestations, and vice versa. As of recent, standard treatment guidelines for PCOS consist of oral contraceptive pills (OCPs) and metformin. While OCPs do address PCOS-related hormonal imbalances, such as elevated testosterone, they do not address the insulin resistance and obesity that are commonly associated with PCOS. As such, metformin has been a standard treatment used to address insulin resistance associated with PCOS, but it is often inadequate, especially in patients with PCOS with refractory obesity and insulin resistance. Women may not get diagnosed with PCOS until they are trying to conceive and facing infertility, weight management difficulties, and hormonal imbalance. This results in significant physical and psychological impacts to women of childbearing age, including acne, hirsutism, menstrual irregularities, weight gain, anxiety, and depression [1,2].

One of the most common manifestations of PCOS is the presence of insulin resistance. Patients with PCOS are 50% more likely to develop insulin resistance when compared to healthy women. Patients with PCOS with insulin resistance possess a higher prevalence of metabolic syndrome, obesity, and type II diabetes mellitus (T2DM) [3]. The presence of insulin resistance in patients with PCOS may indirectly contribute to increased body mass index (BMI) and hyperandrogenism [3-5]. Patients with PCOS often present with normal glucose levels but hyperinsulinemia, which, over time, results in a decrease in sensitivity of insulin signaling. Impaired insulin signals contribute to an increase in oxidative stress, inflammatory cytokines, and abnormal lipid metabolism that will subsequently result in weight gain, metabolic syndrome, and type II diabetes mellitus if left untreated [5].

There is conflicting literature on the most appropriate treatment modalities for patients with PCOS. The most recent guidelines recommend a combination of oral contraceptive pills, such as estradiol-progesterone, and metformin [6]. Recent literature has explored incretin mimetics such as glucagon-like peptide-1 (GLP-1) receptor agonists, designed for T2DM and other insulin-resistant disorders, as adjunct therapy for patients with PCOS. GLP-1 receptor agonists act to reduce insulin resistance by increasing glucose transporters, which in turn decreases inflammation, reduces oxidative stress, modulates lipid metabolism, promotes insulin secretion, slows gastric emptying, and inhibits macrophage secretion of inflammatory cytokines in insulin-dependent tissues. GLP-1 receptor agonists are recommended in the treatment of PCOS because of the relationship between PCOS and other insulin resistance-related clinical manifestations, such as uncontrolled weight gain, high BMI, and unstable lipid panels [4,7,8]. Improving insulin sensitivity in patients with PCOS may lead to improvement in androgen levels and ovarian function, which will lead to a reduction in infertility and dysmenorrhea, symptoms that patients with PCOS commonly experience [4,7,8].

Currently, there are two classes of incretin mimetics that are Food and Drug Administration (FDA)-approved for the management of T2DM and obesity [9]. The first class includes drugs that selectively bind and upregulate the GLP-1 receptor, such as semaglutide, exenatide, liraglutide, and dulaglutide [9]. The second class targets both the GLP-1 receptor and gastric inhibitory peptide (GIP) for upregulation [9]. When GIP is upregulated, insulin and glucagon levels are secreted during periods of hyperglycemia and hypoglycemia, respectively [9]. Tirzepatide belongs to the second class of incretin mimetics and has added therapeutic benefits for patients, such as improved total cholesterol, triglycerides, and low-density lipoprotein (LDL) cholesterol with improved cardiovascular outcomes, such as reduced incidence of advanced cardiovascular disease [8-11]. Most recently, a triple GLP-1 receptor agonist, retatrutide (currently in phase 3 of drug trials), has shown even further improvements in BMI and body weight reduction in obese patients [12,13].

Incretin mimetics have substantial scientific evidence for their therapeutic potential in managing T2DM and insulin-resistant disorders. The aim of this review is to investigate the potential use and benefit of prescribing GLP-1 receptor agonists for patients with PCOS. This review explores the usage of GLP-1 receptor agonists as combination therapy with metformin and as monotherapy for patients with PCOS, and the subsequent metabolic and hormonal effects of the pharmacological therapy, such as improved lipid panels and reduced hyperandrogenism.

## Review

The aim of this review is to analyze current literature on the role of GLP-1 receptor agonists in the management of PCOS. This review focuses on the application of GLP-1 receptor agonists as monotherapy and in combination in the management of insulin resistance in patients with PCOS. This review highlights the metabolic and anthropometric changes seen in patients with PCOS following pharmacological interventions with a GLP-1 receptor agonist.

Search strategy

**Figure 1:**
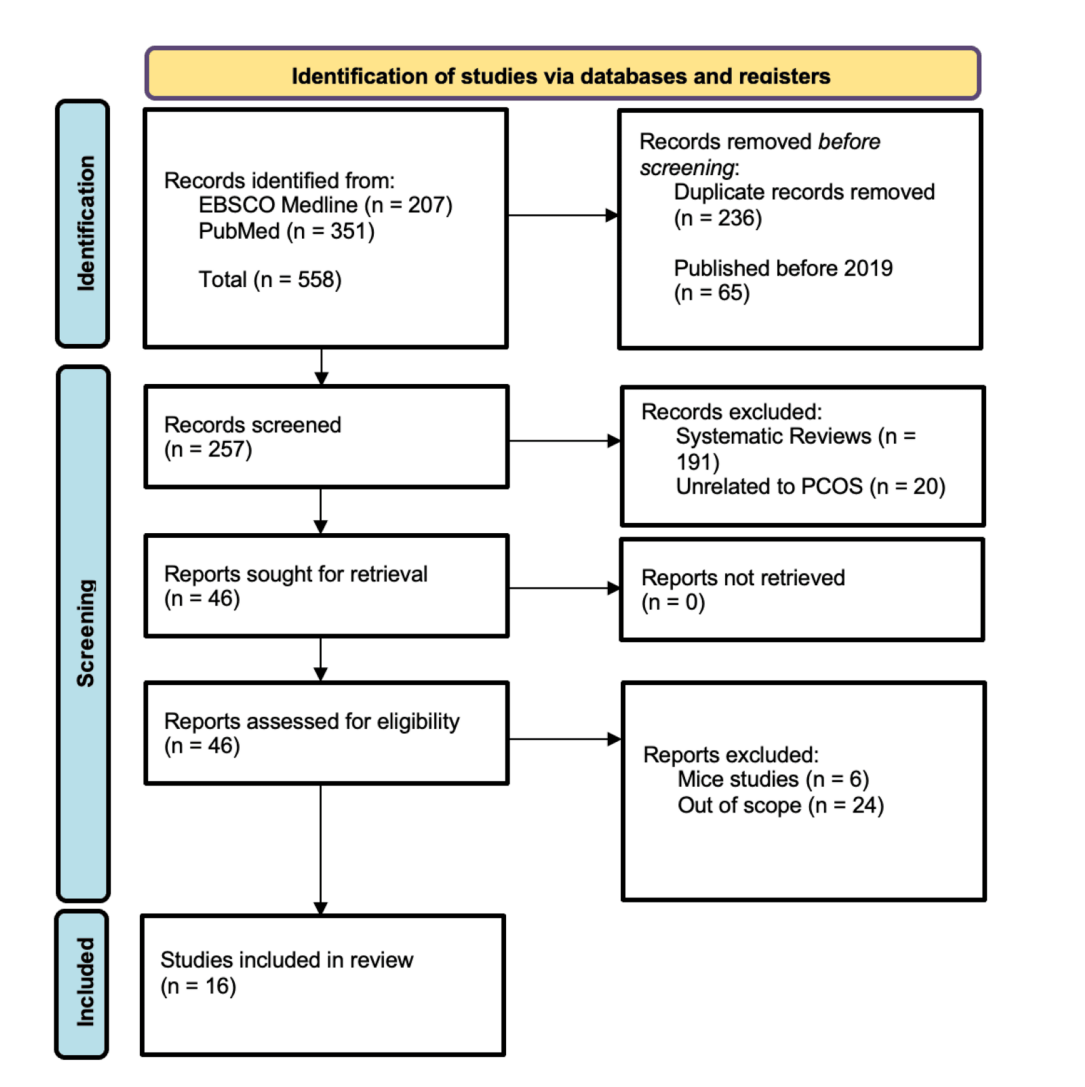
PRISMA diagram detailing the search strategy utilized for article inclusion PRISMA: Preferred Reporting Items for Systematic Reviews and Meta-Analyses, PCOS: polycystic ovarian syndrome

The databases EBSCO Medline and PubMed were utilized in the search for articles for inclusion in this review. The terms “semaglutide AND PCOS,” “tirzepatide AND PCOS,” “tirzepatide AND insulin resistance,” “GLP-1 receptor agonist AND reproductive disorders,” “GLP-1 receptor agonist AND PCOS,” and “retatrutide” were used. These searches yielded a total of 558 articles identified by three reviewers. A total of 16 articles were included for analysis in this review following removal of duplicates and application of inclusion and exclusion criteria. Exclusion criteria included articles written prior to 2019, case studies, case reports, and those otherwise out of scope for the purpose of this review. Figure 1 provides a diagram detailing our selection of articles for review utilizing the Preferred Reporting Items for Systematic Reviews and Meta-Analyses (PRISMA) flowchart.

GLP-1 Receptor Agonists in Combination With Metformin

In a single-center retrospective study, Sassin et al. aimed to compare the effects of pharmacological management of weight loss for 143 patients with PCOS [10]. Researchers compared the efficacy of oral metformin treatment alone to the combination therapy of oral metformin with subcutaneous tirzepatide. Utilizing the Cox proportional hazards regression model, researchers analyzed changes in the weight and BMI of the participants. Researchers found that the participants who received a combination therapy of oral metformin with subcutaneous tirzepatide were twice as likely to experience weight loss as their counterparts who were treated with oral metformin alone [10].

Ma et al. used a similar study design examining 50 patients with PCOS and a BMI greater than 25 kg/m^2^ to study differences using combination therapy with metformin and a GLP-1 receptor agonist compared to metformin alone [14]. The 50 participants were separated into two treatment groups: one group received 500 mg oral metformin three times daily in conjunction with 2 mg weekly exenatide, while the other treatment group received 500 mg metformin alone. Both treatment groups demonstrated improvements in BMI and waist circumference, although the combination therapy demonstrated greater efficacy than metformin alone [14]. Those who received the combination therapy saw a statistically significant decrease in fasting plasma glucose and improvement in insulin response to an oral glucose tolerance test (OGTT) (p<0.050) [14]. Both treatment groups demonstrated changes in lipid panels, including significant improvements in triglycerides, high-density lipoprotein cholesterol (HDL-C), and apolipoprotein A1 (ApoA1) levels, and a significant decrease in total testosterone [14].

The prospective randomized controlled trial by Liao et al. aimed to compare the efficacy between oral metformin (1500 mg) in combination with a hormonal oral contraceptive, cyproterone acetate/ethinyl estradiol (CPA/EE) (2 mg/36 μg), and in combination with a subcutaneous GLP-1 receptor agonist, liraglutide (1.2-1.8 mg), for the pharmacological management of overweight patients with PCOS [6]. This 12-week trial followed 60 patients with PCOS aged 18-50 years old, with a BMI greater than 24 kg/m^2^, diagnosed via the Rotterdam criteria. While both treatments were effective in lowering HbA1c and luteinizing hormone (LH) levels, the group that received the combination therapy of metformin with a GLP-1 receptor agonist had greater reductions in BMI, body weight, waist circumference, fasting blood glucose, and ovulation [6]. Participants in the metformin with CPA/EE group had a greater decrease in hyperandrogenemia than those in the metformin with the GLP-1 receptor agonist group [6].

Xing et al. similarly aimed to observe the effects of oral metformin (1000 mg) monotherapy in comparison to a combination therapy of oral metformin with subcutaneous liraglutide (1.2 mg) on gonadal and metabolic profiles [15]. This randomized controlled trial consisted of 52 patients with PCOS aged 18-40 years old. All patients had hyperandrogenism, ovulatory dysfunction, and a BMI greater than 24 kg/m^2^. None of these patients were on other medications that may have affected insulin sensitivity during the trial. Following the 12-week trial, the participants were assessed for menstrual changes by calculating the number of bleeds, changes in their gonadal profiles by measuring serum LH and follicle-stimulating hormone (FSH), and their response to an oral glucose tolerance test. The results demonstrated that both groups had improvements with their menstrual cycles and metabolism, resulting in a 52% and 88% improvement in menstrual cycle recovery rates in the metformin and combination groups, respectively [15]. However, participants who received the combination treatment had decreases in testosterone, LH, follicle-stimulating hormone (FSH), and progesterone when compared to those who received metformin alone [15].

In a randomized controlled study conducted by Long et al., similar correlations were observed between the combination therapy of oral metformin (0.85 mg) with a subcutaneous GLP-1 receptor agonist liraglutide (0.6 mg) and improved reproductive biomarkers [16]. A 12-week trial with 102 obese or overweight patients diagnosed with PCOS found significant reductions of testosterone, androstenedione, and free androgen index (FAI) following combination therapy with metformin and liraglutide. Their results also demonstrated a statistically significant decrease in participants’ weight, BMI, and waist-to-hip ratio. Additionally, the researchers also noted increases in cardiovascular metrics, including HDL-C and ApoA1, improving lipid metabolism and overall cardiovascular function [16].

A total of 153 prediabetic patients with PCOS were followed for 12 weeks to assess the effectiveness of subcutaneous exenatide (10-20 μg) and oral metformin (1500-2000 mg) for the clinical management of PCOS in a prospective controlled study by Tao et al. [17]. Participants were diagnosed as prediabetic via an oral glucose test and were then randomly placed into three groups: the first group received a daily injection of exenatide, the second group consumed a daily dose of metformin, and the third group received a combination therapy of exenatide and metformin daily. Following treatment, researchers found a statistically significant increase in the rates of sustained remission of prediabetes in both the exenatide-only and combination therapy groups when compared to the metformin-only group [17]. As insulin sensitivities during fasting and the OGTT were comparable across the treatment groups, researchers were able to discern the difference in remission rates across the treatment groups due to the enhancement of postprandial insulin secretion levels by exenatide [17].

An alternative use of metformin in conjunction with a GLP-1 receptor agonist in the management of overweight patients with PCOS was studied. In a two-year observational study, Jensterle et al. aimed to assess whether adding oral metformin after discontinuation of subcutaneous semaglutide would halt weight regain [18]. Patients were treated with 1 mg weekly of semaglutide for 16 weeks, which was then discontinued after 16 weeks and replaced with 2000 mg/day of metformin and lifestyle intervention. BMI and weight loss were then measured two years after cessation of semaglutide and continuation of the metformin treatment. After 16 weeks of semaglutide treatment, there was a statistically significant decrease in weight from 101 kg to 92 kg. After two years of metformin intervention and semaglutide discontinuation, there was no statistically significant increase in weight, which only increased by 3 kg (from 92 kg to 95 kg) [18]. This result suggests that metformin did not result in a significant increase in weight regain after discontinuation of semaglutide; rather, it attenuated weight regain. Similarly, BMI after 16 weeks of semaglutide was significantly decreased from 36.4 kg/m^2^ to 33.5 kg/m^2^, and after two years of metformin intervention, BMI only increased from 33.5 kg/m^2^ to 34.4 kg/m^2^, which was not statistically significant. Trends were similar for testosterone levels. Testosterone significantly decreased after 16 weeks of semaglutide treatment from 6.16 ng/dL to 4.12 ng/dL and did not increase after stopping semaglutide and managing with metformin for two years [18].

GLP-1 Receptor Agonists as Individual Therapy

The use of semaglutide as an intervention for patients with PCOS was further tested in human participants. In a randomized controlled trial, Jensterle et al. studied 20 patients who were diagnosed with PCOS and obesity, with an average BMI of 37 kg/m^2^ [19]. The patients were randomly assigned to either a placebo or a subcutaneous semaglutide group. The test group received eight weekly injections of subcutaneous semaglutide at a dose of 0.25 mg for the first two weeks, followed by 0.5 mg for two weeks, and finally 1 mg for four weeks. In the semaglutide group, they found a 37% greater retention in stomach contents, a significant decrease in body weight and body circumference, lower levels of androstenedione and free testosterone, and a reduction in glucose and HbA1c values compared to their baseline numbers [19]. However, in this study, the differences were not found to be statistically significant between the test group and the control group [19].

In an observational study, Carmina and Longo assessed the implications of semaglutide alone as a treatment for PCOS in human participants [7]. Twenty-seven obese patients with PCOS who originally failed to lose weight with exercise and lifestyle changes were treated with 0.5 mg of subcutaneous semaglutide weekly for three months [7]. After treatment with semaglutide, BMI, body weight, insulin, and Homeostatic Model Assessment for Insulin Resistance (HOMA-IR) values significantly decreased [7]. All of the subjects who received semaglutide injections saw improvements in their fasting blood glucose, with as many as 80% of the subjects returning to a normal fasting blood glucose range. At least a 5% decrease in body weight was seen in 21 of the 27 patients, which indicated they were responsive to the treatment. Twenty-one responsive patients completed an additional three months of treatment, during which they saw additional overall weight loss; however, there was no further improvement in their fasting glucose, insulin, or HOMA-IR [7].

The use of subcutaneous exenatide on cardiovascular biomarkers in patients with PCOS was studied in an observational study conducted by Dawson et al. [8]. The participants were administered 5 ug of exenatide twice daily for one month, followed by 10 ug twice daily for three months. There was no observed effect on total cholesterol, high-density lipoprotein (HDL), or LDL; however, improvements were observed in triglycerides (from 1.3 to 1.2 mmol/L) [8]. No change was observed in glucose or insulin levels, but C-reactive protein (CRP) improved from 8.5 mg/L to 5.6 mg/L. The study also demonstrated a modest improvement in weight loss in patients with PCOS who received intervention with exenatide, as well as improvements in serum markers involved in endothelial function, inflammation, and clot function [8].

Tang et al. conducted a randomized controlled trial regarding the effectiveness of pharmacological intervention with a similar GLP-1 agonist, exenatide [20]. Thirty-two women diagnosed with PCOS with a BMI greater than 23 kg/m^2^ received a subcutaneous injection of exenatide twice daily (started at 5 ug and then increased to 10 ug after one month) for three months in addition to regular exercise and dietary guidance. The participants were then compared before and after treatment against 35 healthy counterparts that were of a normal BMI and had no history of menstrual cycle irregularity. The researchers found that prior to pharmacological intervention, the PCOS subjects had a higher waist-to-hip ratio, systolic blood pressure, HbA1c, and fasting blood glucose levels [20]. Additionally, in their laboratory results, they had elevated triglycerides, LDLs, and branched-chain amino acids. Following treatment with exenatide, researchers found an improvement in the metabolites, in particular the metabolism of branched-chain amino acids [20].

Ninety-two women diagnosed with obesity and PCOS were assigned either to a treatment group with 3 mg of subcutaneous liraglutide or a placebo group for 32 weeks to assess the effects of liraglutide on their body weight and hyperandrogenism in a randomized controlled trial conducted by Elkind-Hirsch et al. [21]. In the test group, there was a significant decrease in body weight from 111 kg to 104 kg after two weeks [21]. The mean BMI decreased from 41.6 kg/m^2^ to a mean BMI of 39.1 kg/m^2^. There were statistically significant decreases in waist circumference, HOMA-IR, and FAI. There were additional improvements seen in the test group, including in the patient’s hyperandrogenism and menses occurrence, whereas in the control group, those factors were unchanged [21].

Comparison of Different GLP-1 Receptor Agonists

There are several studies that compare the efficacy of different GLP-1 receptor agonists on insulin resistance. However, there are no current studies that compare GLP-1 receptor agonists in the context of patients with PCOS. In the randomized controlled trial by Mather et al., researchers examined 117 adults 20-74 years of age, all diagnosed with type II diabetes mellitus (T2DM), with a BMI between 25 and 45 kg/m^2^ over the course of a 28-week trial [9]. The participants were randomly separated into three groups: the first group received a weekly subcutaneous tirzepatide injection of 15 mg, the second group received a weekly semaglutide injection of 1 mg, and the third group received a placebo. Researchers found that the participants who received tirzepatide had a significantly greater reduction in fasting glucose levels when compared to those who received semaglutide [9]. There was also a significant reduction in insulin secretion rate (ISR) in the tirzepatide group versus the semaglutide group, indicating improvement in insulin sensitivity. While both groups resulted in significantly reduced glucagon, tirzepatide administration resulted in a greater reduction. Overall, this study demonstrated that the administration of tirzepatide resulted in greater glycemic control compared to semaglutide due to its improvements in insulin secretion rate, as well as reduced glucagon and sensitivity to insulin [9].

In a similar randomized controlled trial, Heise et al. examined 117 patients diagnosed with T2DM for at least six months who were administered either 15 mg subcutaneous tirzepatide, 1 mg subcutaneous semaglutide, or a placebo for 28 weeks to assess HbA1c and fasting insulin [22]. HbA1c in the tirzepatide group improved by 22 mmol after 28 weeks, compared to 17.9 mmol in the semaglutide group [22]. Body weight in the tirzepatide group decreased by 11.2 kg, compared to a 6.9 kg decrease in the semaglutide group. While both GLP agonists were effective at improving HbA1c and insulin levels, tirzepatide demonstrated greater overall glycemic efficacy when compared to semaglutide injections [22].

The most recent development of a triple GLP-1 receptor agonist known as retatrutide shows even further improvements in insulin sensitivity, weight loss, and glycemic control [14]. Jastreboff et al. conducted a phase 2, double-blind, randomized, placebo-controlled trial with 338 patients who either had a BMI of 30-50 kg/m^2^ or 27-30 kg/m^2^ with at least one weight-related condition [12]. The participants were randomly assigned to either receive subcutaneous retatrutide in a variety of doses or a placebo. Weight changes were assessed after 24 weeks and 48 weeks. After 24 weeks, the groups that received the smallest dose (1 mg) and the largest dose (12 mg and 2 mg initial dose) had a decrease of 7.2% and 17.5% in body weight, respectively [12]. After 48 weeks, the groups that received the smallest dose (1 mg) and the largest dose (12 mg and 2 mg initial dose) had a decrease of 8.7% and 24.2% change in body weight, respectively. Retatrutide treatment administered over a 48-week period resulted in significant reductions in body weight, BMI, and waist circumference when compared to the control group [12].

Rosenstock et al. conducted a randomized, double-blind controlled trial with 281 patients diagnosed with T2DM prescribed either 1.5 mg dulaglutide, 0.5 mg retatrutide, 4 mg (starting dose: 2 mg) retatrutide, 4 mg (no escalation) retatrutide, 8 mg (starting dose: 2 mg) retatrutide, 8 mg (starting dose: 4 mg) retatrutide, 12 mg (starting dose: 2 mg) retatrutide once weekly via subcutaneous injection, or placebo [13]. HbA1c and body weight were measured from baseline, at 24 weeks, and at 36 weeks. Changes in body weight after 36 weeks were significant compared to placebo and 1.5 mg dulaglutide in all groups who were administered retatrutide, except for the 0.5 mg retatrutide group [13]. Improvements in insulin levels were significant in the 8 mg (starting dose: 4 mg) and 12 mg (starting dose: 2 mg) retatrutide groups. At 24 weeks, HbA1c decreased significantly in all retatrutide groups from baseline. After 36 weeks, there was a significant decrease in HbA1c compared to 24 weeks in all retatrutide groups, except for the 0.5 mg group [13].

Table 1 summarizes the primary research articles included in this review.

**Table 1:** Summary table of the 16 articles in the review This table summarizes all 16 articles reviewed and includes the article study design, data collection performed, aim of each study, findings, and limitations noted. BMI: body mass index, CPA/EE: cyproterone acetate/ethinyl estradiol, CRP: C-reactive protein, FAI: free androgen index, FSH: follicle-stimulating hormone, GIP: gastric inhibitory polypeptide, GLP-1: glucagon-like peptide-1, HbA1c: hemoglobin A1c, HDL: high-density lipoprotein, HOMA-IR: Homeostatic Model Assessment for Insulin Resistance, ISR: insulin secretion rate, LDL: low-density lipoprotein, LH: luteinizing hormone, MET: metformin, PCOS: polycystic ovarian syndrome

Article	Patients	Study design	Summary of intervention	Outcome	Risks/limitations
Liao et al. (2024) [6]	60 overweight patients with PCOS aged 18-50 years with a BMI of >24 kg/m^2^	Single-center prospective randomized controlled trial	A 12-week trial compared the efficacy of oral metformin (1500 mg) in combination with a hormonal oral contraceptive, CPA/EE (2 mg and 35 ug, respectively), versus in combination with a subcutaneous GLP-1 receptor agonist, liraglutide (1.2-1.8 mg).	While both groups were effective in lowering HbA1c and LH levels, the group that received combination therapy of metformin with a GLP-1 receptor agonist had greater reductions in BMI, body weight, waist circumference, fasting blood glucose, and ovulation.	Relatively small sample size and single-center design; additionally, lifestyle changes were not controlled for, as the researchers aimed to isolate the effects of the drug on overweight patients with PCOS
Carmina and Longo (2023) [7]	27 patients with PCOS and obesity who originally failed to lose weight with lifestyle modifications	Observational study	Patients were treated with 0.5 mg of subcutaneous semaglutide weekly for three months.	After treatment with semaglutide, BMI, body weight, insulin, and HOMA-IR values significantly decreased (p<0.01). All subjects who received semaglutide injections saw improvements in their fasting blood glucose. Twenty-one patients experienced a 5% decrease in body weight, deeming them responsive. These patients then completed an additional three months of treatment, during which they saw additional overall weight loss; however, there was no further improvement in their fasting glucose, insulin, or HOMA-IR.	Small sample size
Dawson et al. (2019) [8]	30 obese/anovulatory women diagnosed with PCOS	Observational study	Participants were administered 5 ug of exenatide twice daily for one month, followed by 10 ug twice daily for three months.	There was no observed effect on total cholesterol, HDL, or LDL; however, improvements were observed in triglycerides (from 1.3 to 1.2 mmol/L). No change was observed in glucose or insulin levels, but CRP improved from 8.5 mg/L to 5.6 mg/L. The study also demonstrated a modest improvement in weight loss in patients with PCOS who received intervention with exenatide, as well as improvements in serum markers involved in endothelial function, inflammation, and clot function	High drop-out rate of participants due to the side effects of the medication (nausea and vomiting); lack of controls
Mather et al. (2024) [9]	117 adults aged 20-74 diagnosed with type II diabetes mellitus with a BMI between 25 and 45 kg/m^2^	Multicenter randomized controlled trial	Participants were randomly separated into three groups: the first group received a weekly subcutaneous tirzepatide injection of 15 mg, the second group received a weekly semaglutide injection of 1 mg, and the third group received a placebo for 28 weeks.	The participants who received tirzepatide had a significantly greater reduction in fasting glucose levels when compared to those who received semaglutide (p<0.01). There was also a significant reduction in ISR in the tirzepatide group versus the semaglutide group, indicating improvement in insulin sensitivity (p<0.01). While both groups resulted in significantly reduced glucagon, tirzepatide administration resulted in a greater reduction (p<0.01). Overall, this study demonstrated that the administration of tirzepatide resulted in greater glycemic control compared to semaglutide due to its improvements in ISR, as well as reduced glucagon and sensitivity to insulin.	Small sample size; at the time, only 1 mg of semaglutide was available, so effects on higher doses were unknown
Sassin et al. (2023) [10]	143 overweight/obese patients with PCOS	Single-center, retrospective, prospective randomized controlled trial	Electronic medical records of patients were evaluated. At the first visit, their weight and BMI were evaluated. Forty-six non-diabetic patients were treated with MET alone, and 30 non-diabetic patients were treated with MET + tirzepatide (COMBO). Changes in weight, BMI, and percentage of starting weight were analyzed using the Cox proportional hazards regression model.	Weight loss was around two times higher in patients with PCOS taking COMBO treatment with MET and a GLP-1/GIP receptor agonist compared to MET alone.	Further prospective randomized controlled studies are needed to validate findings and assess clinically related metabolic and fertility outcomes
Jastreboff et al. (2023) [12]	338 patients who either had a BMI of 30-50 kg/m^2^ or 27-30 kg/m^2^ with at least one weight-related condition	Multicenter double-blind, randomized, placebo-controlled trial	Participants were randomly assigned to receive either subcutaneous retatrutide in a variety of doses or a placebo. Weight changes were assessed after 24 weeks and 48 weeks.	After 24 weeks, the groups that received the smallest dose (1 mg group) and the largest dose (12 mg and 2 mg initial dose) had a decrease of 7.2% and 17.5% in body weight, respectively. After 48 weeks, the groups that received the smallest dose (1 mg group) and the largest dose (12 mg and 2 mg initial dose) had a decrease of 8.7% and 24.2% change in body weight, respectively. Retatrutide treatment administered over a 48-week period resulted in significant reductions in body weight, BMI, and waist circumference when compared to the control group (p<0.01).	The racial and geographic homogeneity of the sample suggests that the results may not be generalizable to the population
Rosenstock et al. (2023) [13]	281 patients with type II diabetes mellitus	Multicenter double-blind, randomized, placebo- and active-controlled trial	Patients received either placebo, 1.5 mg dulaglutide, 0.5 mg retatrutide, 4 mg (starting dose: 2 mg) retatrutide, 4 mg (no escalation) retatrutide, 8 mg (starting dose: 2 mg) retatrutide, 8 mg (starting dose: 4 mg) retatrutide, or 12 mg (starting dose: 2 mg) retatrutide once weekly via subcutaneous injection. HbA1c and body weight were measured from baseline, at 24 weeks, and at 36 weeks.	Changes in body weight after 36 weeks were significant compared to placebo and 1.5 mg dulaglutide in all groups who were administered retatrutide, except for the 0.5 mg retatrutide group (p<0.001). Improvements in insulin levels were significant in the 8 mg (starting dose: 4 mg) and 12 mg (starting dose: 2 mg) retatrutide groups (p=0.0082 and p<0.001, respectively). At 24 weeks, HbA1c decreased significantly in all retatrutide groups from baseline (p<0.001). After 36 weeks, there was a significant decrease in HbA1c compared to 24 weeks in all retatrutide groups except for the 0.5 mg group (p<0.001).	Relatively small sample and a homogenous population sample
Ma et al. (2021) [14]	50 women diagnosed with PCOS with a BMI of >25 kg/m^2^	Single-center retrospective study	The 50 participants were randomly separated into two treatment groups for a 12-week pharmacological intervention. Those in the first treatment group received an oral metformin dose of 500 mg three times daily in combination with a once-weekly injection of 2 mg exenatide, while the second treatment group received oral 500 mg metformin alone.	Combination therapy was more effective than metformin alone in reducing body weight, BMI, and waist circumference, and improving insulin sensitivity in overweight/obese women with PCOS, with minimal side effects.	Small sample size, open-label design, and short study duration limit the results of this study
Xing et al. (2022) [15]	52 patients with PCOS aged 18-40 years with a BMI of >24 kg/m^2^	Single-center prospective randomized controlled trial	12-week trial aimed to compare the effects of oral metformin (1000 mg) monotherapy in comparison to combination therapy of oral metformin with subcutaneous liraglutide (1.2 mg) on gonadal and metabolic profiles. Following the 12-week trial, participants were assessed for menstrual changes by calculating the number of bleeds, changes in their gonadal profiles by measuring serum LH and FSH, and their response to an oral glucose tolerance test.	Both groups had improvements with their menstrual cycles and metabolism, resulting in a 52% and 88% improvement in menstrual cycle recovery rates in the metformin and combination groups, respectively. Participants who received the combination treatment had decreases in testosterone, LH, FSH, and progesterone when compared to those who received metformin alone.	Single-center design, relatively small sample size, and short treatment duration
Long et al. (2023) [16]	102 obese/overweight patients with PCOS aged ≥18 years with a BMI of ≥24 kg/m^2^	Single-center prospective randomized controlled trial	Patients were treated with dinae-35, a low dose of liraglutide (0.6 mg once daily), and metformin (0.85 mg twice daily) for 12 weeks.	After 12 weeks of liraglutide in combination with metformin, there was a statistically significant decrease in weight in participants, with an average 7.25 kg decrease in weight (p<0.001). There was also a statistically significant decrease in BMI after the 12 weeks; there was a 2.89 decrease in BMI (p<0.001). There was a statistically significant decrease in the waist-to-hip ratio from 0.90 to 0.88 (p<0.001).	Possible selection bias, recall bias, and the ignorance of the influence of lifestyle on weight loss
Tao et al. (2021) [17]	153 prediabetic patients with PCOS	Single-center prospective randomized controlled trial	Patients were followed for 12 weeks to assess the effectiveness of subcutaneous exenatide (10-20 ug) and oral metformin (1500-2000 mg). Participants were then randomly placed into one of the following three treatment groups: the first group received a daily injection of exenatide, the second group consumed a daily dose of metformin, and the third group received a combination therapy of exenatide and metformin daily.	There was a statistically significant increase in the rates of sustained remission of prediabetes in both the exenatide-only and combination therapy groups when compared to the metformin-only group (p=0.003 and p=0.027, respectively).	Single-center design, relatively small sample size, and short duration of drug treatment
Jensterle et al. (2024) [18]	25 women with PCOS and obesity	Two-year observational study	Patients were treated with 1 mg weekly of semaglutide for 16 weeks, which was then discontinued after 16 weeks and replaced with 2000 mg/day of metformin and lifestyle intervention. BMI and weight loss were then measured two years after cessation of semaglutide and continuation of the metformin treatment.	After 16 weeks of semaglutide treatment, there was a statistically significant decrease in weight from 101 kg to 92 kg (p<0.001). After two years of metformin intervention and semaglutide discontinuation, there was no statistically significant increase in weight; weight only increased by 3 kg (from 92 kg to 95 kg). BMI after 16 weeks of semaglutide was significantly decreased from 36.4 kg/m^2^ to 33.5 kg/m^2^ (p<0.001), and after two years of metformin intervention, BMI only increased from 33.5 kg/m^2^ to 34.4 kg/m^2^, which was not statistically significant. Testosterone significantly decreased after 16 weeks of semaglutide treatment from 6.16 ng/dL to 4.12 ng/dL (p=0.012) and did not increase after stopping semaglutide and managing with metformin for two years.	Small sample size and absence of a control group
Jensterle et al. (2023) [19]	20 patients with PCOS and obesity	Single-center randomized controlled trial	The patients were randomly assigned to either a placebo or a subcutaneous semaglutide group. The test group received eight weekly injections of subcutaneous semaglutide at a dose of 0.25 mg for the first two weeks, followed by 0.5 mg for two weeks, and finally 1 mg for four weeks	In the semaglutide group, they found a 37% greater retention in stomach contents, a significant decrease in body weight and body circumference (p=0.005 and p=0.018, respectively), lower levels of androstenedione and free testosterone, and a reduction in glucose and HbA1c values compared to their baseline numbers.	Small sample size
Tang et al. (2019) [20]	32 women with PCOS with a BMI of >23 kg/m^2^	Single-center randomized controlled trial	Patients received a subcutaneous injection of exenatide twice daily (started at 5 ug and then increased to 10 ug after one month) for three months in addition to regular exercise and dietary guidance. The participants were then compared before and after treatment against 35 healthy counterparts who were of a normal BMI and had no history of menstrual cycle irregularity.	The researchers found that prior to pharmacological intervention, the PCOS subjects had a higher waist-to-hip ratio, systolic blood pressure, HbA1c, and fasting blood glucose levels. Following treatment with exenatide, researchers found an improvement in triglycerides, LDLs, and branched-chain amino acids.	Small sample size
Elkind-Hirsch et al. (2022) [21]	92 women diagnosed with obesity and PCOS	Single-center randomized controlled trial	Patients were assigned either to a treatment group with 3 mg of subcutaneous liraglutide or a placebo group for 32 weeks to assess the effects of liraglutide on their body weight and hyperandrogenism.	In the test group, there was a significant decrease in absolute body weight from 111 kg to 104 kg after two weeks (p=0.02). There were statistically significant decreases in waist circumference, HOMA-IR, and FAI (p=0.011, p=0.05, and p=0.006, respectively). There were additional improvements seen in the test group, including in the patient’s hyperandrogenism and menses occurrence, whereas in the control group, those factors were unchanged.	Absence of gold-standard measures of insulin sensitivity and a high drop-out rate
Heise et al. (2022) [22]	117 patients diagnosed with type II diabetes for at least six months	Multicenter, randomized controlled trial	Patients were administered either 15 mg subcutaneous tirzepatide, 1 mg subcutaneous semaglutide, or a placebo for 28 weeks to assess HbA1c and fasting insulin.	HbA1c in the tirzepatide group improved by 22 mmol after 28 weeks, compared to 17.9 mmol in the semaglutide group. Body weight in the tirzepatide group decreased by 11.2 kg, compared to a 6.9 kg decrease in the semaglutide group. While both GLP agonists were effective at improving HbA1c and insulin levels, tirzepatide demonstrated greater overall glycemic efficacy when compared to semaglutide injections.	Differences in the duration of exposure at the highest dose for the two agents evaluated; all patients were also being treated with metformin, which may have underestimated the insulin sensitivity effect of tirzepatide

Discussion

Analysis of the available literature for the use of incretin mimetics in conjunction with traditional metformin therapy suggests that these medications may be an effective adjuvant treatment for patients with PCOS concerned with reducing body weight, BMI, and waist circumference. Multiple studies demonstrated a reduction in HbA1c and insulin levels of patients with PCOS who received monotherapy and combination therapy with a GLP-1 receptor agonist [6,7,10,14,16,17]. Some researchers also noted improvements in cardiovascular biomarkers, such as HbA1c and insulin, and lipid panels, such as HDL and triglycerides, after combination therapy with GLP-1 receptor agonists and metformin [8,14,20]. Additionally, some studies demonstrated improvements in hyperandrogenism, such as improved menstruation and reduction in hirsutism, as well as cystic morphology, such as a reduction in ovarian cysts, following pharmacological management of patients with PCOS with a GLP-1 receptor agonist [15,18,19,21]. Moreover, these results were not worsened following two-year termination of the GLP-1 receptor agonist if metformin was used as maintenance therapy [18]. GLP-1 receptor agonists as a monotherapy in PCOS treatment suggest similar benefits.

The few studies that compared the efficacy of semaglutide and tirzepatide to each other demonstrated that tirzepatide may be a more effective treatment option for patients with PCOS due to the greater reductions in body weight, BMI, and waist circumference [9,22]. Tirzepatide also showed greater improvements in insulin sensitivity rate compared to semaglutide. Tirzepatide injections demonstrated greater glycemic efficacy than semaglutide injections, with significantly greater decreases in HbA1c [9,22]. The most recent development of a triple GLP-1 receptor agonist known as retatrutide demonstrated even further improvements in insulin sensitivity, weight loss, and glycemic control [12,13]. Retatrutide’s significant improvements in insulin sensitivity and weight reduction could prove it to be a potentially useful adjuvant in the management of patients with PCOS, and this warrants further research.

A major strength of this review was the replicability of the studies and the quality of the research studies that investigated GLP-1 receptor agonists in the PCOS population. The current literature regarding incretin mimetics as therapy for patients with PCOS revealed a significant number of studies regarding the use of semaglutide and liraglutide specifically, as well as their use in combination with metformin. Because of the large number of studies that used similar pharmacological interventions and study designs, we were able to make stronger inferences from the data. Additionally, all these studies share a common theme of reduction in body weight and improved insulin sensitivity among patients, confirming that these medications could be promising for patients with PCOS who exhibit similar clinical findings, including increased weight gain, dysmenorrhea, and an increased number of ovarian cysts. This review was limited due to the low quantity of literature regarding GLP-1 receptor agonists as a treatment for PCOS. While the available literature proves valuable in quality to this review, the sheer amount of literature regarding the topic is limited. As a result, some of the articles used in this review focused on the use of GLP-1 receptor agonists for obesity and insulin resistance rather than PCOS specifically. Due to obesity and insulin resistance being a common manifestation of PCOS, this review was able to identify a possible link in using GLP-1 receptor agonists to treat these manifestations in PCOS. Additionally, this review was limited due to the current literature available examining the use of tirzepatide as a specific intervention for patients with PCOS and the comparison of effectiveness between the classes of incretin mimetics. Additionally, since retatrutide is still undergoing FDA trials, we were unable to draw significant conclusions about their specific implications for PCOS, rather the potential benefits for the population, which could be determined by reviewing improvements in laboratory data and clinical symptoms.

While incretin mimetics all suggest promising improvements in insulin sensitivity, BMI, and body weight for patients with PCOS, the exact mechanism behind how these medications affect dysmenorrhea and androgen imbalance associated with PCOS is unclear. While some studies show that normal menses returns after semaglutide treatment, it is ambiguous as to whether this is secondary to reductions in body weight or improved insulin sensitivity post-treatment. Further research should be conducted to determine the mechanism behind how the use of incretin mimetics affects the appearance of polycystic ovaries and hyperandrogenism in patients with PCOS. Furthermore, additional research should be done to assess the long-term effects of incretin mimetics, not only as a whole, but also on maintaining body weight and insulin sensitivity, as well as their effectiveness in improving fertility in patients with PCOS.

## Conclusions

The development of GLP-1 receptor agonists and their proven benefits in improving insulin sensitivity, body weight, and BMI suggest a promising therapy for patients with PCOS due to the overlapping pathophysiology between the condition and the medications’ current approved uses. This review found that patients with PCOS treated with GLP-1 receptor agonists, both as adjuvant therapy and monotherapy, had significant improvements in their anthropometric measurements, as well as biomarkers of insulin resistance. Additionally, a few studies suggested added benefits of improved cardiovascular markers and hyperandrogenism following GLP-1 receptor agonist therapy. While the studies in this review present promising advancements in the management of patients with PCOS, the limited literature investigating the use of GLP-1 receptor agonists as a specific treatment for PCOS imposes difficulty in determining their utilization as either a monotherapy or in conjunction with standard treatments. Additionally, it is difficult to determine whether patients who benefit from GLP-1 receptor agonist therapy are benefiting from treatment of PCOS-related manifestations, such as obesity and insulin resistance, rather than treating the root cause of PCOS.

More research is needed to determine whether GLP-1 receptor agonists may show a link in treating the primary cause of PCOS. The limited literature further demonstrates the complexity of PCOS, both in its pathophysiology and management. However, the suggested insulin-resistant pathophysiology of PCOS suggests that the use of incretin mimetics may provide promising therapeutic benefits for patients with PCOS. Further research should be conducted to specifically compare the effectiveness of GLP-1 receptor agonists for use in patients diagnosed with PCOS to determine which medications might be best utilized in the management of the population. Despite its complexity, the development of GLP-1 receptor agonists proves to be an exciting new horizon in the management of patients with PCOS and might offer researchers a deeper understanding of its pathophysiology. Our hope is that with further investigation, incretin mimetics can be incorporated into treatment guidelines for patients with PCOS, a monumental step that we believe will prove to improve patients’ quality of life and overall health status.
